# Validation and Testing of a Suicide Prevention Program in Preventing Suicidal Ideation and Improving the Mental Well-Being of School-Going Adolescents: Protocol for a Pre-Post Intervention Study

**DOI:** 10.2196/67193

**Published:** 2025-12-12

**Authors:** Yasmin Nadeem Parpio, Rozina Nuruddin, Tazeen Saeed Ali, Nuruddin Mohammed, Uzma Rahim Khan, Salman Shahzad, Murad Moosa Khan, Mehreen Aslam

**Affiliations:** 1 School of Nursing and Midwifery Aga Khan University Karachi Karachi Pakistan; 2 Institute of Clinical Psychology Karachi Pakistan

**Keywords:** prevention program, strategies, intervention, measures, mental well-being, mental health, psychological health, adolescent, teenage

## Abstract

**Background:**

Globally, around 800,000 people die by suicide annually, with 77% of these deaths occurring in low- and middle-income countries. Suicidal ideation, frequently observed among adolescents, is directly linked to suicide attempts. Pakistan has witnessed a marked escalation in suicide rates in recent years, with Gilgit-Baltistan (GB) reporting the highest incidence. Extensive research indicates that tailored suicide prevention strategies can mitigate suicidal ideation, attempts, and related fatalities.

**Objective:**

This study aims to validate and evaluate the efficacy of suicide prevention programs, RAAHI (the guide) and safeTALK, tailored to the cultural and social context of GB, Pakistan, in preventing suicidal ideation and improving the mental well-being of school-going adolescents.

**Methods:**

The investigation will assess the efficacy of the RAAHI intervention among 267 adolescents across 4 private schools in GB using an interrupted time-series design with a pre-post test framework. The first intervention, RAAHI, a suicide literacy module, is designed to empower participants with the knowledge and skills to recognize signs of suicidal ideation, engage them empathetically, and connect individuals in crisis with appropriate support. The second intervention, safeTALK, a 4-hour educational workshop, incorporates presentations, videos, discussions, and interactive sessions to teach participants the tell, ask, listen, and keep safe steps. Outcome measures include changes in knowledge, confidence, willingness to intervene, and help-seeking behaviors, analyzed using descriptive statistics, 2-tailed paired *t* tests, and ANOVA. Ethics approval was granted by the Aga Khan University Ethics Review Committee (2023-8509-24844).

**Results:**

As of April 2025, 267 participants have been recruited from 2 of the 4 schools; data collection commenced in March 2025 and is projected to conclude in August 2025. Final results are expected to be published by February 2026.

**Conclusions:**

This study will offer critical insights into the adaptation and effectiveness of the RAAHI suicide prevention intervention in a low-resource, culturally specific setting. The findings are anticipated to inform scalable suicide prevention initiatives in school settings across Pakistan and similar low- and middle-income contexts, ultimately contributing to reduced suicidal ideation and attempts among adolescents.

**International Registered Report Identifier (IRRID):**

DERR1-10.2196/67193

## Introduction

### Background

Suicide is a notable global public health concern, resulting in a staggering number of fatalities annually [1]. It ranks among the 5 leading causes of death, representing more than one-third of all fatalities [2]. The rising global suicide rate, surpassing 800,000 deaths annually, underscores the urgency of addressing this critical public health concern [3]. In Australia, suicide is the leading cause of death among individuals aged between 15 and 24 years, with an annual rate of 11.6 deaths per 100,000 population [4]. Moreover, each suicide case potentially corresponds with more than 20 attempted suicides, emphasizing the urgent need for suicide prevention interventions. The World Health Organization [3] suggests an alarmingly high prevalence of suicidal ideation, encompassing nonfatal suicide attempts and self-harm. This tragic occurrence is widespread across the globe, with 77% of suicides taking place in low- and middle-income countries (LMICs) [2].

In addition, on a global scale, suicide ranks as the fourth leading cause of death for adolescents and young adults aged between 15 and 29 years [5]. Over the past 5 years, the United Arab Emirates has recorded the highest rate of suicides globally (11.4%), followed by the United Kingdom (10.80%), Nepal (10.33%), the United States (8.9%), and Pakistan (7%) [6]. Within the spectrum of mental health disorders, suicide emerges as the foremost cause of violent death, even among the economically active age group of people aged between 15 and 44 years [6]. According to a meta-analysis, 22.3% of college students at some point in their lives had suicidal thoughts, plans or made attempts [7]. In addition, a survey of 8113 high school students in Hawaii found that 16.4% of the adolescents reported having suicidal thoughts, and nearly 10% acknowledged having attempted suicide at least once in their lives [8].

Adolescents are highly susceptible to mental health problems due to academic stress, interpersonal issues, and the transition to adulthood; all of these factors exert substantial influence on their physical, emotional, social, and mental well-being [9]. In Pakistan, these challenges are exacerbated by social and cultural pressures, mental health being considered a stigma, and limited access to treatment, which impacts social functioning and academic success, with long-term consequences [10]. Evidence indicates that adolescents susceptible to self-harm often refrain from seeking support from parents or professionals; instead, they turn to their peers for assistance [11], who, due to their limited experience and knowledge, are usually not able to provide the required assistance.

Mental well-being signifies flourishing across emotional, psychological, and social domains, characterized by satisfaction, resilience against adversity, a sense of purpose, positive feelings, and the capacity for meaningful connections and activities [12]. Given the significance of mental well-being for adolescents and the concerning prevalence of mental disorders within this demographic, prioritizing mental health interventions tailored to address the unique needs and concerns of this demographic is essential due to the high burden of mental disorders prevailing in this age group [13]. Numerous suicide prevention programs aim at enhancing skills in identifying and supporting individuals at risk while also improving their self-efficacy, mental health literacy, and attitudes [14]. The enhancement of self-efficacy and mental health knowledge is linked to increased resilience in youth, fostering a sense of control, thereby significantly reducing the likelihood of engaging in suicidal ideation [15].

Increasing evidence suggests that school-based suicide prevention interventions are effective in their approach [11]. Such interventions span various levels of prevention, encompassing primary efforts, such as public awareness campaigns; secondary approaches, such as gatekeeper training programs; and tertiary interventions, such as psychotherapy and postvention strategies, including survivor support groups. Similarly, expanding the conventional classification of mental health interventions, suicide prevention strategies can be categorized into universal (targeting the public) or selective (aimed at specific high-risk groups with elevated lifetime risk, such as adolescent and psychiatric inpatients) [16].

A systematic review of 13 randomized controlled trials (RCTs) reported that the risk of suicide-related behavior among youth exposed to an educational intervention was 0.31 times lower compared to those in the unexposed group [17]. Similarly, another review of 14 studies assessing the impact of school-based gatekeeper training revealed enhancements in gatekeepers’ knowledge, attitudes, self-efficacy, and inclination to intervene [18]. An RCT of the Signs of Suicide program showed a decrease in suicide attempts over a 3-month period [19]. Moreover, a large-scale RCT of a Youth Aware of Mental health program, involving more than 11,110 secondary school students across 10 European countries, revealed a 55% reduction in incidents of suicide attempts and a 50% decrease in severe suicidal ideation compared to control groups at 12 months after intervention [20].

In Australia, a crucial component of the integrated, regionally based approach to suicide prevention was gatekeeper training, which was widely implemented as a universal approach in secondary schools. Such training programs aim to equip students with skills to serve as peer gatekeepers, capable of identifying suicide warning signs, addressing concerns, and facilitating appropriate referrals for assistance and care [18,21].

safeTALK, a gatekeeper training initiative, seeks to elevate awareness regarding suicide prevention by framing it as a collective community responsibility, envisioning a helping role for every individual [22]. In addition, it empowers individuals without professional mental health backgrounds to confidently and effectively support someone showing signs of emotional distress. Studies have shown that this training enhances the effectiveness of informal mental health support for those experiencing emotional imbalance [2].

Pakistan, located in Southeast Asia, grapples with numerous health and social challenges due to persistent waves of violence, political instability, and recurrent changes in the social infrastructure, which have significantly increased the burden of physical and mental ailments impacting individuals of all ages [23]. Unfortunately, Pakistan lacks accurate statistics on suicide deaths and suicidal attempts [24] due to underreporting of such cases, which happens because of social factors, particularly the stigma attached to such cases. However, recently published studies provide some insights into suicide deaths in Pakistan, where adolescents exhibited suicidal ideation and attempted suicide at the rates of 40% and 7%, respectively [25]. Moreover, in 2019, suicide mortality rates in Pakistan were estimated to be 8.9 per 100,000 population [26,27]. Gilgit-Baltistan (GB), comprising valleys located among the snow-clad mountains of northern Pakistan, has shown an alarming increase in adolescent mental health issues, including suicidal ideation and suicide rates, which has been particularly exacerbated during and following the COVID-19 pandemic [28].

To address the rising trend of suicidal ideations and attempts, prevention initiatives, such as the safeTALK program, hold significance, as they can help in disseminating awareness and education among adolescents through suicide literacy. Programs such as these aim to cultivate courage and confidence among adolescents to openly discuss suicidal thoughts and ideations and encourage help-seeking behaviors to approach professionals, such as psychologists or counselors. Extensive evidence supports the efficacy of the safeTALK program, which has been successfully implemented in various countries [29]. However, Pakistan, with its unique cultural and social setup, has yet to conduct a comprehensive analysis of the effectiveness of such programs.

The RAAHI suicide prevention program for school-going adolescents in GB was based on the theoretical frameworks of mental health literacy and resilience theory. Mental health literacy refers to the information and views about mental disorders that help with their detection, care, and prevention [30]. Improving mental health literacy enables people to recognize indicators of mental distress, cultivates good attitudes about getting help, and raises awareness of available support resources. By including mental health literacy in our program, we hope to provide teenagers with the tools to recognize and treat mental health concerns proactively. Self-determination theory states that when students feel autonomous and competent in their learning environment, they create a strong sense of academic productivity. According to the social cognitive theory [31], developing self-efficacy through supportive teaching improves learning ability, mediating the beneficial relationship between perceived teaching style and academic resilience [32]. Emotional dysregulation, defined as difficulties in understanding and managing emotions, is a substantial risk factor for adolescents’ mental health concerns [33]. Cyberbullying involves repetitive harassment that can occur anytime, spreading swiftly to a huge audience, with anonymity magnifying its negative effects. Forms include defamation, harassment, and threatening communications, which have psychological and social effects for survivors, such as increased feelings of isolation, anxiety, sadness, and even suicide [34]. Adolescence is a phase marked by turmoil and upheaval, rendering individuals more prone to psychological disorders. These episodes often begin as fleeting but can become enduring, leading to psychiatric conditions, diminished performance, escalated medical expenses, and an elevated risk of subsequent self-harm. Global data indicate that the incidence of online harassment varies between 14.6% and 52%, while rates of being targeted by harmful digital acts range from 6.3% to 32%. The anonymity and widespread nature of cyberbullying can intensify emotional distress, potentially initiating or exacerbating suicides in susceptible youth [35]. Mindfulness significantly mediates the relationship between cyberbullying and cognitive emotion regulation among adolescents.

Resilience theory investigates how people respond positively to adversity, focusing on variables that facilitate recovery from difficult situations. In the context of suicide prevention, resilience refers to protective characteristics that assist individuals in navigating suicidal situations [36]. It includes a positive attributional style and a sense of purpose in life that protect people from suicidality during stressful circumstances [37]. Building resilience includes learning life skills, including problem resolution, emotional regulation, and effective coping mechanisms [38]. By incorporating resilience-building components into this intervention, we want to improve adolescents’ ability to handle stress and minimize their vulnerability to suicidal thoughts and behaviors.

National and international suicide prevention strategies emphasize the significance of schools for targeted efforts [39], as they are conducive and accessible settings for implementing such programs. Leveraging the educational environment effectively enables reach and engagement with adolescents, thereby enabling the provision of relevant support and resources to mitigate the risk of suicidal ideation and behavior. Hence, this study intends to validate and assess the effectiveness of RAAHI (the guide) and a translated and contextualized version, named safeTALK, of the suicide prevention program in GB. Programs such as safeTALK can help lower teenager suicides by teaching them about suicide, giving them the confidence to talk about suicidal feelings, and encouraging them to seek help from professionals such as psychologists or counselors.

There is sufficient evidence that the safeTALK program is efficient and has been implemented in many other countries [29]. This study is the first of its kind in Pakistan to rigorously evaluate the impact of a suicide prevention program. Given the country’s increasing suicide rates and the unique cultural and socioeconomic factors that influence mental health, such as stigma and limited access to services, this research is crucial for addressing a critical public health concern [40]. There is a notable scarcity of culturally adapted, evidence-based suicide prevention programs in Pakistan. Existing interventions often do not consider the unique sociocultural dynamics of Pakistani society, leading to less effective outcomes. Hence, this study aims to evaluate the effectiveness and contextual relevance of suicide prevention programs (RAAHI and safeTALK) in reducing suicidal ideation and enhancing mental well-being among school-going adolescents in GB. In addition, it examines how these programs influence suicide literacy, confidence in discussing suicide, willingness to seek help, mental health outcomes, adaptive coping strategies, and self-efficacy, ensuring their applicability within the local sociocultural context.

### Research Questions

The primary research question about suicidal ideation is as follows: What is the effect of suicide prevention programs (RAAHI and safeTALK) in reducing suicidal ideation among school-going adolescents in GB?

The secondary research question is as follows: What is the effect of suicide prevention programs (RAAHI and safeTALK) among school-going adolescents in GB on (1) perceived suicidal awareness; (2) perceived Social Support Questionnaire; (3) suicide literacy; (4) attitude toward seeking help related to suicidal ideation; (5) life attitude schedule; (6) depression, anxiety, and stress level; (7) adaptive coping strategies; and (8) self-efficacy.

### Null Hypotheses

The hypotheses are as follows:

- Hypothesis 1—suicidal ideation risk among school-going adolescents in GB is similar at baseline and 8 weeks following the implementation of the suicide prevention program.
- Hypothesis 2—Compared to baseline, there is no mean difference in suicide awareness, suicide literacy, and attitude to seek help after 8 weeks of participation in a suicide prevention program (RAAHI and safeTALK) among school-going adolescents.
- Hypothesis 3—Compared to baseline, there is no difference in the mean score of depression, adaptive coping strategies, and self-efficacy after 8-week suicide prevention programs (RAAHI and safeTALK) among school-going adolescents.

## Methods

The protocol was prepared in accordance with the SPIRIT (Standard Protocol Items: Recommendations for Interventional Trials) guidelines [41]. The trial has been registered in ClinicalTrials.gov (NCT06721364).

### Study Design

This research will use a single-group quasi-experimental pre-post interrupted time-series design to allow for examining any change in the levels of the dependent variables before and after the administration of the intervention [42]. Moreover, there will be no control group, as it would be unethical to withhold intervention from potentially distressed adolescents [42]. The study design allows for assessing intervention effects over multiple time points; it is particularly suitable in settings where randomization is not feasible [43]. To control confounding variables, statistical adjustments and sensitivity analyses will be used to account for potential biases, such as maturation effects and historical events. In addition, the inclusion of multiple assessment points (time point 1, time point 2, and time point 3) strengthens internal validity by capturing trends over time rather than relying on a single before or after measure. By addressing these methodological considerations, we enhance the study’s rigor and reliability.

### Study Phases

**Figure 1:**
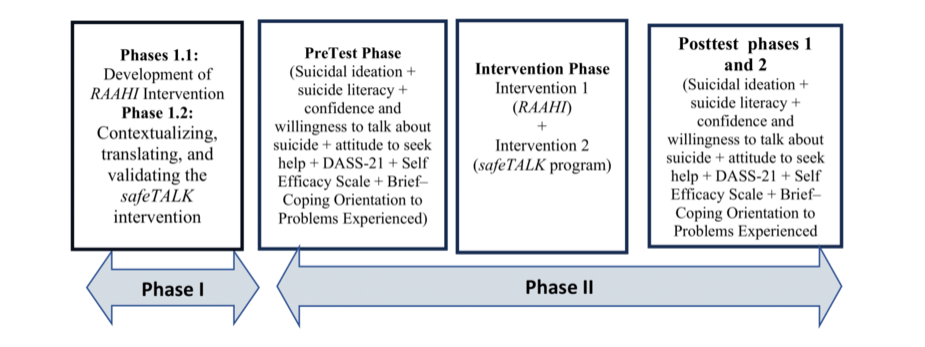
Phases of the study.

This study will be carried out in 2 phases, from January 30, 2024, to October 30, 2025 (Figure 1). Phase 1 will comprise developing the RAAHI intervention by the researcher, followed by translation of the safeTALK intervention module, ensuring its contextualization and validating it through expert review. The effectiveness of the intervention will be evaluated in phase 2 using a quasi-experimental pre-post interrupted time-series design. RAAHI focuses on comprehensive suicide literacy and fostering open discussions to combat stigma about suicide, incorporating wellness education, while safeTALK is a shorter, more targeted workshop teaching specific intervention skills and immediate crisis responses using the tell, ask, listen, and keep safe (TALK) steps to emphasize immediate practical skills for identifying and addressing suicidal behavior by reducing depression and anxiety and improving adaptive coping strategies and the self-efficacy level.

### Phase 1

#### Phase 1.1: Development of the RAAHI Module

During phase 1A, an interdisciplinary team comprising mental health nurses, psychologists, and educationists will create the RAAHI module for implementation. The objectives, content, timing, and activities for each module will be designed (Table 1). Experts in the field of mental health will then examine the material for clarity and appropriateness.

**Table 1:** Intervention module objectives and duration.

Session or week and suicide prevention intervention modules		Objectives	Duration (h)	
**RAAHI** **intervention**				3
	Module 1: circle of connection	Establish a warm and inclusive environment through an engaging ice-breaking activity, promoting a sense of connection among participants		
	Module 2: common misconceptions and facts	Establish a warm and inclusive environment through an engaging ice-breaking activity, promoting a sense of connection among participants		
	Module 3: warning signs for suicide	Provide a comprehensive overview of suicide warning signs, empowering participants to recognize and understand these indicators for prompt intervention		
	Module 4: protective factors	Provide a holistic overview of protective factors against suicide, fostering understanding and encouraging the creation of a supportive and nurturing environment		
	Module 5: coping strategies	Offer practical coping strategies, empowering participants with tools to manage negative thoughts, navigate challenges, and build resilience for improved emotional well-being		
**safeTALK intervention**				4
	Module 1: introduction of safeTALK	Establish core beliefs about suicide and its prevention		
	Module 2: importance of safeTALK	Recognize individuals experiencing suicidal thoughts Link individuals with suicidal thoughts to appropriate suicide first aid resources		
	Module 3: Introduce steps of TALK^a^	Present standard procedures for safeTALK, including community and personal reasons, introducing TALK steps, which seek to increase people’s self-awareness and help-seeking behavior while reducing suicidal ideation		

^a^TALK: tell, ask, listen, and keep safe.

#### Phase 1.2: Translation of the safeTALK Intervention Module and Contextual Validation

Translation of the RAAHI and safeTALK intervention material into Urdu, using the backward translation approach, and validation by experts will be carried out to make it culturally and contextually relevant. A team of mental health nurses, clinical psychologists, psychiatrists, and members of the Brain and Mind Institute at the Aga Khan University in Karachi, Pakistan, will validate the intervention module (Table 1).

### Research Setting

GB, located in northern Pakistan, boasts spectacular mountain scenery, as it is spread over a region that has 2 mountain ranges: the Karakoram and the Himalayan. Although the region has great natural beauty, it grapples with poverty, limited mental health services, and harsh living conditions. Efforts to improve well-being in GB require addressing these challenges through initiatives. This study will be conducted in 4 private schools located in northern Pakistan. The rationale for selecting these schools is chiefly the readiness of the school management to implement, adopt, and sustain the intervention. Because this intervention will be used in the Pakistani context for the first time, the willingness of the school principals and their flexibility in mobilizing resources will play a major role. Although the setting will limit external validity, these study sites will be purposively selected, as they will facilitate assessing the feasibility of the intervention in a Pakistani context [44].

### Study Population

#### Inclusion Criteria

Both male and female adolescents enrolled in grades 9 and 10 who can comprehend English and Urdu will be included. In addition, adolescents and their parents must provide consent to participate in the study.

#### Exclusion Criterion

Adolescents who will be absent or sick on the day of data collection and intervention will not be included in the study.

### Sampling Strategy

A multicenter experimental study using consecutive sampling will be conducted to examine the effectiveness of school-based preventive interventions for suicidal behavior. Four schools in the GB regions will be recruited. Eligible schools will be randomly selected. Within the selected schools (clusters), all students in grades 9 and 10 will be invited to participate. This approach of inviting all eligible students within the selected clusters aims to enhance representativeness and minimize potential selection bias that could arise if only specific students were chosen from within each school. This is the best technique to explore the phenomena and to gain rich data from the participants [42]. The chosen design enables the detection of trends and changes over time. This method aids in distinguishing intervention effects from other temporal variables to observe whether changes correlate with the intervention time, which strengthens causal inferences. Multiple assessments establish a baseline trend, differentiating natural development from intervention-induced changes [45]. Regular data collection allows for adjustment of external events influencing outcomes. Despite lacking a control group, this approach enables each participant to serve as their own control, enhancing causal inference [46].

### Sample Size

The sample size for this study was calculated using G*Power (version 3.1.9.2; Heinrich Heine University Düsseldorf) and Microsoft Excel, applying the paired sample formula. Data from the study by Bailey et al [47,48] reported pre- and postintervention knowledge scores with mean values of 16.88 (95% CI 16.17-17.19) and 20.88 (95% CI 20.36-21.41), respectively. To convert the CI into SD, statistical calculations were performed using the standard normal distribution and t-distribution, yielding SD values of approximately 2.2 for preintervention and 1.2 for postintervention scores. With a significance level (α) of .05, power (1–β) of 80%, an estimated effect size of 1.2, and a correlation coefficient of 0.5, the required sample size was determined to be 223 participants. Accounting for a 20% dropout rate [49], the final sample size was adjusted to 267 school-going adolescents to ensure sufficient statistical power for detecting meaningful differences in knowledge scores.

### Recruitment of Participants

#### Step 1

Participants will be recruited from 4 private schools under the Aga Khan Education Service network using consecutive sampling. Approval will be obtained from the school principals, followed by coordination with the administration to arrange a designated space for study activities.

#### Step 2

A recruitment flyer will be distributed to students in grades 9 and 10. Interested students will be instructed to contact their parents to obtain initial verbal consent before participation.

#### Step 3

Parents will be provided with study details in a private setting. The researcher will explain the study’s purpose, procedures, and expectations, addressing any questions. Written informed consent will be obtained for eligibility screening, intervention participation, and pre- and posttest assessments. To ensure transparency, parents will also receive a copy of the consent form for further review.

#### Step 4

Eligible students will be identified based on predefined criteria. Those who meet all eligibility requirements will be enrolled in the study.

To mitigate low participation rates, multiple outreach efforts, including follow-up meetings, will be conducted. In cases where logistical constraints arise, such as scheduling conflicts or space limitations, alternative arrangements (eg, rescheduling sessions or using available classrooms) will be made to facilitate smooth study implementation.

### Procedure

#### Intervention 1: RAAHI

RAAHI is a suicide literacy module that is specially designed to provide individuals with essential knowledge and skills by equipping them with tools to navigate the complexities of mental health and suicide prevention. This module aims to empower people to recognize signs of suicidal ideation, engage in empathetic communication through open discussion, combat stigma, and connect individuals in crisis with appropriate support and resources. The duration of the module will be 3 hours. Hence, RAAHI seeks to create a world where suicide literacy is widespread, compassion is the key to preventing tragedy, and no one feels isolated in their struggle, thereby contributing to a more empathetic and supportive future (Table 1).

#### Intervention 2: safeTALK

The safeTALK program is an educational initiative designed for suicide prevention among adolescents, consisting of a 4-hour workshop that incorporates presentations, videos, discussions, and questions. The workshop is designed according to the standard practices of safeTALK to assist participants in (1) identifying warning signs of suicide; (2) avoiding frequent impulses to overlook, dismiss, or avoid discussions about suicide; (3) recognizing and responding to situations where suicidal thoughts may be present by applying fundamental TALK steps; and (4) connecting individuals contemplating suicide with first aid assistance and additional community resources [2]. The skill training program concentrates on bolstering adolescents’ understanding of activities that promote mental well-being, fostering resilience, honing distress management abilities, and enhancing their capacity to identify early signs of suicidal ideation or behavior both within themselves and among others. The program also equips participants with strategies for suicide crisis intervention.

The intervention involves the components mentioned subsequently.

### Phase 2: Testing the Effectiveness of the Suicide Prevention Program

#### Pretest Phase

Students willing to participate will be asked to provide their consent by signing a form or paper before starting the pen-and-paper pretraining survey. Students will be explicitly informed that participation is entirely voluntary, and their decision to participate or not to participate will not affect their training in any way. First, the psychologist and the researcher will conduct a risk assessment of the students for any previous history or current complaints of suicidal ideation and behavior. A 40- to 60-minute window will be allocated before the training commences to enable participants to complete the pretest survey. In addition, the level of suicide literacy will also be evaluated through validated and self-report questionnaires. Along with this, students’ level of confidence and willingness to discuss suicidal ideation and attitudes toward seeking help from others, adopting coping abilities, and self-efficacy level will also be assessed. Following the pretest phase, the students will enter the intervention phase.

#### Intervention

The intervention phase consists of 2 workshops in which the students will be required to participate. The first workshop consists of 2 hours of RAAHI intervention which will provide them with general knowledge about suicide, risk factors, consequences, and preventive strategies; this will help participants improve their suicide literacy. Moreover, a booklet comprising different activities will also be provided to participants during the training. In addition, the second session will introduce the 4-hour safeTALK program, which seeks to increase people’s self-awareness and help-seeking behavior while reducing suicidal ideation. This is a culturally relevant program as it applies basic TALK steps and connects the person with suicidal thoughts with suicide first aid help and further community resources. Moreover, a pocket card containing the “suicide alert steps” will also be provided to the participants at the end of the training. In this study, the safeTALK workshops will be delivered to adolescents, approximately 30 students at a time. The researcher, along with a psychologist, will administer this training with the help of translated videos and material in Urdu, which is culturally appropriate and has been modified and adapted from LivingWorks.

#### Posttest Phase

**Figure 2:**
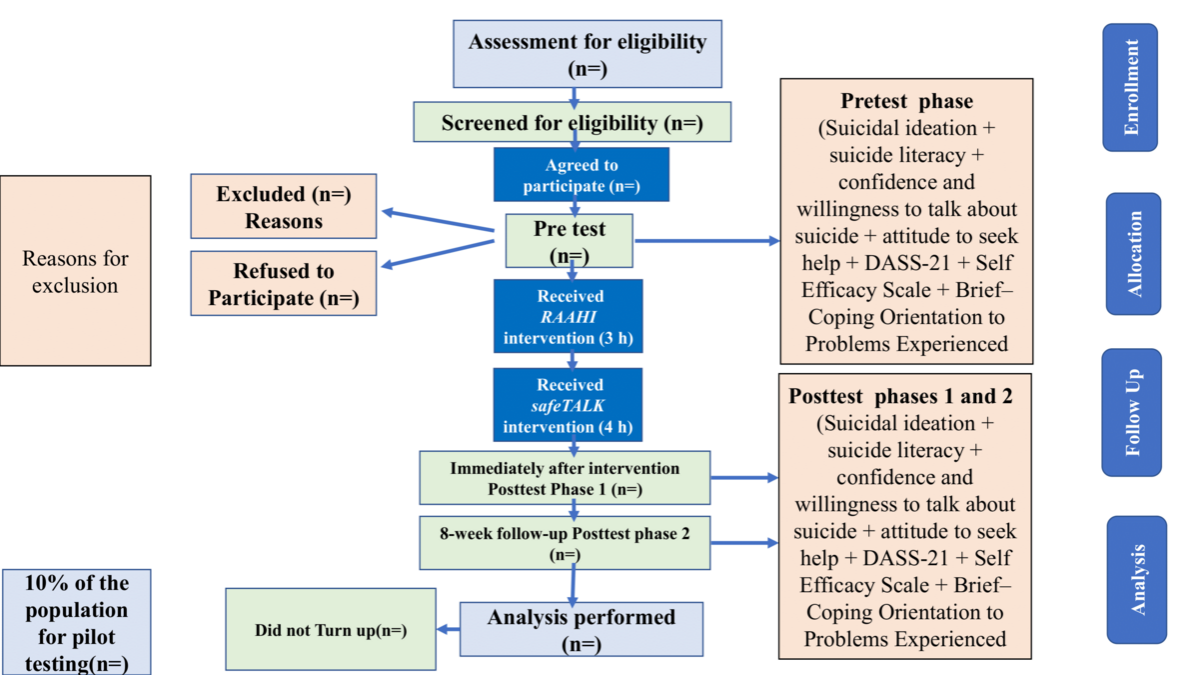
CONSORT (Consolidated Standards of Reporting Trials) diagram for participant flow.

The data will be recollected on all outcome measures during posttest phase 1, which is right after the intervention, and posttest phase 2, which will be after 8 weeks of the intervention (Figure 2).

### Data Collection Tools

A predesigned survey will be administered to individuals who express interest in participating in this study and willingly agree to provide the relevant information. The questionnaire consists of 3 sections. The first section will gather detailed demographic information and include eligibility questions, followed by the Modified Scale for Suicidal Ideation (MSSI) by Miller et al [43]. Its translated version is available as it was used in a study of suicide in Pakistan [50]. The second section includes questions about suicide literacy, confidence, and willingness to talk about suicide, followed by an attitude to seek help. In the last section, depression will be assessed using the Depression, Anxiety, and Stress Scale–21 Items (DASS-21), along with questions about the ability to adopt coping skills and self-efficacy.

### Study Outcomes

#### Suicidal Ideation

The level of suicide literacy among the participants will be measured through the MSSI by Miller et al [43], comprising 21 items. It was translated and used in Urdu by Bakht et al [51]. The MSSI has shown strong internal consistency, with a Cronbach α coefficient ranging from 0.87 to 0.94 [52]. The severity of suicidal ideation, as assessed by the MSSI total score, is categorized into 3 levels. A total score ranging from 0 to 8 indicates a low level of suicidal ideation. In contrast, a total score between 9 and 20 suggests a moderate level of suicidal ideation. These adolescents may experience more frequent or persistent thoughts of suicide, potentially accompanied by feelings of hopelessness or despair. Finally, a total score of 21 or higher reflects a severe level of suicidal ideation, indicating a significant risk of self-harm or suicide. Adolescents in this category may have intense and intrusive thoughts of ending their life, and immediate intervention and support are often necessary to ensure their safety. Severe suicidal ideation is indicated by a total MSSI score exceeding 20, coupled with an intense suicidal intention, represented by a score of more than 1 on item 7. In addition, an active desire to make an attempt is signaled by a score exceeding 1 on item 3, while a serious plan for suicide is indicated by a score of more than 1 on item 15.

#### Perceived Suicide Awareness

In total, 14 items of the Perceived Suicidal Awareness Scale from a previous suicide prevention study were used to evaluate perceived suicide awareness [2,48]. The original Perceived Suicidal Awareness Scale consists of 5 items assessing perceived knowledge about suicide and help-seeking resources, 3 items measuring willingness to discuss suicide and seek assistance, 5 items evaluating confidence in discussing suicide and obtaining help, and 1 item examining the intention to seek help. Responses were recorded on a 5-point scale, ranging from 0 (strongly disagree) to 4 (strongly agree). The scale was translated into Urdu and subsequently back translated into English. Any inconsistencies were identified, deliberated upon, and resolved. The finalized version was piloted with the target population. This scale has shown strong internal consistency, with a Cronbach α coefficient ranging from 0.87 to 0.94. Questions will be asked to determine the participant’s level of confidence while discussing issues regarding suicide. The questionnaire will use a Likert scale that will range from 1 to 3, where 1 will indicate “disagree,” whereas 3 will be read as “agree.” The scale was adopted from the studies by Kinchin et al [2] and Baggio et al [53]. The suicide skills confidence subscale (Cronbach α=0.84) assesses respondents’ confidence in working with clients with suicidal thoughts in regard to training, skills, comfort, and supervision. Possible scores range from 4 to 20, with higher scores indicating greater confidence. The questionnaire will also focus on determining the participants’ willingness to talk about suicide and suicidal ideation with others. To analyze the willingness, the statements will be assessed on a Likert scale ranging from 1 to 3.

#### Perceived Social Support Questionnaire

The Social Support Questionnaire was adapted from the Perceived Social Support (Fragebogen zur Sozialen Unterstützung) 6-item scale developed by Kliem et al [54] and Lin et al [55]. This German tool evaluates adolescents’ capacity to recognize the availability of assistance from friends, family, and peers in times of need. For this study, minor adjustments were made to the questionnaire with the authors’ permission while preserving its core purpose. The scale demonstrated high reliability, with an internal consistency (Cronbach α) of 0.90.

#### Suicide Literacy

Suicide literacy, a vital component of mental health literacy, encompasses knowledge of suicide’s warning signs, causes, risk factors, and preventive measures [56]. The Literacy of Suicide Scale, developed and evaluated by McGillivray et al [20], includes 26 items assessing suicide literacy across 4 dimensions: signs and symptoms (5 items), causes and triggers (10 items), risk factors (7 items), and treatment and prevention (4 items) [56]. Each question has a correct answer, scored as 1 point, while incorrect or “I don’t know” responses score 0. In this study, 1 question was removed, resulting in a 25-item scale with scores ranging from 0 to 25, where higher scores reflect better suicide literacy.

#### Attitude Toward Help-Seeking Behavior

The Attitude Toward Seeking Professional Psychological Help Scale by Fischer and Farina [57] will be used to assess participants’ attitudes toward seeking help regarding mental health problems. This tool consists of 10 items rated on a Likert scale ranging from 1 (disagree) to 4 (agree). The total score, obtained by summing individual item scores, reflects a positive attitude toward seeking psychological help. With reliable psychometric properties, the instrument has a reliability index ranging from 0.80 to 0.86 [58].

#### Life Attitudes Schedule

The Life Attitudes Schedule–Short Form by Rohde et al [59] is a 24-item binary measure assessing thoughts and behaviors associated with an increased risk of future suicide ideation, attempts, and completions. Participants respond with either true (1) or false (0), and the scores are summed up from a scale score ranging from 0 to 24 to create a total scale score. Higher scores reflect greater engagement in life-enhancing behaviors, while lower scores indicate increased participation in life-threatening behaviors. The scale has exhibited commendable internal consistency, with a Cronbach α of 0.78 [60], and demonstrated excellent construct validity with regard to history of suicide attempts, depressive symptoms, and impulsivity [61].

#### Depression, Anxiety, and Stress Level

The depression, anxiety, and stress levels will be assessed by the DASS-21 questionnaire, which was translated into Urdu, the national language of Pakistan, by Gillani et al [62], to assess the perceived stress levels and psychological well-being of the Pakistani population in Islamabad. Its internal consistency was assessed, revealing a Cronbach α of 0.94. DASS-21 will serve as a screening tool, evaluating stress, depression, and anxiety over the past week through 21 items, each consisting of a set of 7 questions. Responses on the Likert scale range from 0 to 3, where “never” corresponds to 0, “sometimes” to 1, “frequently” to 2, and “always” to 3. [63]. Depression, anxiety, and stress levels of participants will be assessed using predefined cutoff values as follows: depression score of less than 6, anxiety score of less than 5, and stress score of less than 9, in accordance with the DASS-21 guidelines [64].

#### Adaptive Coping Strategies

The Brief–Coping Orientation to Problems Experienced questionnaire, a validated 28-item self-report tool developed by Carver [65], assesses both effective and ineffective coping strategies in response to stressful life events. Participants rate their engagement in each strategy on a scale from 1 (not doing at all) to 4 (doing a lot). The questionnaire encompasses 14 coping strategies, including self-distraction, active coping, denial, substance use, emotional support, instrumental support, behavioral disengagement, venting, positive reframing, planning, humor, acceptance, religion, and self-blame. Scores for each coping style range from 2 to 8, with higher scores indicating a greater inclination toward using that specific coping mechanism [66]. The tool has demonstrated an internal reliability of 0.86 [67].

#### Self-Efficacy

Self-efficacy will be measured using a 10-item Generalized Self-Efficacy Scale, which gauges individuals’ optimistic beliefs about their competence, encompassing determination, problem-solving, and coping skills [68]. Responses range from 1 (not at all true) to 4 (exactly true). The Urdu translation was already done by Mansoor and Ahmad [69].

### Feedback and Evaluation

At the conclusion of the workshop, the participants will be provided with a feedback questionnaire to freely share their thoughts about this session as well as comments on suicidal ideation. This feedback questionnaire has been adapted and modified from a previous study conducted in Pakistan [70]. All participants will be asked to complete a feedback questionnaire assessing several aspects on a Likert scale. These include the comprehensibility of the language used, relevance of the content, enjoyment of the session, effectiveness of strategies used, usefulness of the session, clarity and simplicity of activities, excellence of the overall training, practicality of the session, appropriateness of its duration, and inviting comments and suggestions for improvement [71].

### Reliability and Validity of Tools

The appropriateness and relevance of the tool in the Pakistani context will be checked by experts. Psychologists will assess the validity through the validation of the content for clarity and relevance. The tools will be translated into Urdu by a linguistic expert. The Urdu versions of the questionnaires, alongside the English version, will be distributed to 4 mental health experts for assessment of language clarity, word choice and order, and representativeness of the construct. Feedback provided by the experts will be integrated into the questionnaires. In addition, back translation into English will be conducted by 4 experts. The content validity will be measured by following a standard protocol of content validity index (CVI) scores.

As mentioned by Yusoff [72], the researcher will seek definitive evaluation evidence for the tool. A rubric will be used, with a Likert scale, to assess relevance (1=not relevant, 2=somewhat relevant, 3=pretty relevant, and 4=very relevant) and clarity (1=not clear, 2=somewhat clear, 3=quite clear, and 4=very clear). Ratings of 1 and 2 will prompt consideration for potential adjustments, while evaluations of 3 and 4 will be considered acceptable. The evaluation panel will make recommendations for alterations, resulting in a clarity score of 0.95 and a relevance score of 0.86, according to the CVI analysis. Moreover, for determining the tool’s reliability, Cronbach α will be used. Pilot testing, involving 10% (4/40) of the sample size, will be conducted to ensure reliability. The resulting CVI and Cronbach α coefficient will provide statistical confirmation regarding the reliability, validity, and clarity of the study questionnaire.

### Data Collection

The principal investigator has received safeTALK training from LivingWorks and will train data collectors for the data collection process. The training includes presentations, videos, discussions, questions, and role-play. All participants will receive identical training at no expense, with no alterations in the delivery or content. Demographic information will be solely collected from participants who provide consent and agree to complete both pre- and posttraining surveys; however, the analysis will be restricted to data furnished by those who complete the surveys based on per-protocol analysis [73]. To promote data quality, study personnel will be trained in the administration of these instruments, and procedures for double data entry, verification, and management will be implemented to ensure accuracy and completeness. Data collection forms will be securely stored, with access limited to authorized personnel to maintain confidentiality [74]. Strategies to promote participant retention and complete follow-up will include clear communication about the study’s importance and duration during the consent process, maintaining regular contact with participants and ensuring the study procedures are convenient and acceptable. For participants who discontinue or deviate from intervention protocols, efforts will be made to collect available outcome data to allow for intention-to-treat analysis, where feasible.

### Time Frame for Data Collection

Each participant will receive a short questionnaire at 3 points in time: pretest baseline data (T1), immediately after the intervention (T2), and 8 weeks after the intervention (T3). This self-filled questionnaire will collect data on suicidal ideation, level of suicide literacy, level of confidence, and openness to disclose suicidal ideas to others. The researcher will be responsible for checking for and verifying any missing data with participants, which will help to identify and correct errors in the datasets.

### Data Analysis

Statistical analysis will be conducted using SPSS (version 26; IBM Corp). The normality of data distribution will be assessed, and continuous variables will be summarized using means and SDs (for symmetrical distributions) or medians and IQRs (for skewed distributions), while categorical variables will be presented as frequencies and proportions. Changes in continuous variables (suicidal awareness, suicide literacy, and help seeking) over time will be analyzed using a linear mixed-effects model, and the binary outcome of current suicidal ideation will be analyzed using a population-averaged generalized estimating equation. Linear regression will be used to estimate β coefficients and 95% CIs for predictor variable effects. To compare pre- and postintervention scores on the Literacy of Suicide Scale, a paired samples 2-tailed *t* test will be used, and repeated measures ANOVA with a Bonferroni post hoc test will also be calculated. All primary and exploratory findings will include effect sizes, CIs, and significance levels. While exploratory subgroup analyses may be conducted, they will be clearly identified and interpreted cautiously due to their post hoc nature. Any deviations from the original analysis plan will be justified, and results will be presented with transparency in well-organized tables and figures, including appropriate titles, legends, and footnotes. Data distribution will be further assessed using histograms and the Shapiro-Wilk test to confirm normality assumptions, and the homogeneity of variances will be verified using the Levene test. Any anomalies in the data will be discussed in relation to potential methodological or contextual factors.

### Data Monitoring and Management

Data monitoring and management for this study, coordinated by the School of Nursing and Midwifery at the Aga Khan University, with strategic guidance from a steering committee, involves several key roles and processes. Trained psychologists will collect data, while the primary author will oversee data management (entry validation, cleaning, and secure storage) and monitor participant safety during data collection using safeTALK training, with protocols for immediate counselor referrals if distress occurs. A dedicated, separate data analyst will conduct statistical analyses. An independent data monitoring committee, composed of external experts, including clinicians and a biostatistician operating under a charter, will periodically review trial data for safety, efficacy, and overall conduct, managing interim analyses with predefined stopping guidelines and restricted access to results. Comprehensive procedures are in place for managing and reporting adverse events, with investigators responsible for the timely reporting of serious events. Finally, independent personnel will conduct periodic audits, detailed in a separate plan, to ensure trial quality and compliance.

### Ethical Considerations

#### Overview

This research is fundamentally guided by a commitment to ethical principles, ensuring participant protection. This study has received formal approval from the Aga Khan University Ethics Review Committee (AKU-ERC; 2023-8509-24844) and is transparently registered on ClinicalTrials.gov (NCT06721364). Should any significant adjustments to the study protocol arise (such as changes to eligibility, outcomes, or analysis), these will be communicated efficiently to the investigators, AKU-ERC, the trial registry, and other relevant parties (such as participants or journals) using an online portal.

#### Ethics Approval and Consent to Participate

The interventions in this study strictly adhere to the principles and procedures specified in the Declaration of Helsinki. All experimental protocols have been thoroughly reviewed and approved by the AKU-ERC (Multimedia Appendix 1). Informed consent will be obtained from all study participants, ensuring ethical standards are followed throughout the research process. Transportation incentives will be provided for participation.

#### Consent for Publication

Participants will be asked for their consent during the data collection process, explaining that the collected data may be used for the dissemination of scientific knowledge. However, no personal names or specific identifying information will be revealed in the published material to ensure confidentiality and privacy (Multimedia Appendix 2). Participants will also be requested to give permission to publish any relevant data or materials without disclosing their identity.

Participation in this study is entirely voluntary. The research team, composed of psychologists and the principal investigator, will secure informed consent from parents or guardians after clearly explaining the study’s objectives during sessions at the school, allowing ample time for inquiries and ensuring a pressure-free decision environment. Students will provide their own informed assent (Multimedia Appendix 3). It is crucial that participants know they can withdraw from the study at any stage without facing any disadvantage; should they choose to withdraw, their collected information will be removed from the study analysis. Currently, no ancillary studies requiring separate consent for data use are planned.

Maintaining participant confidentiality is a priority throughout the research process—before, during, and after data collection. Personal information will be gathered securely, and anonymity will be protected by using unique identification codes and pseudonyms, effectively separating personal identities from research data. All electronic data files are password protected, and any reports generated will exclude identifying details such as names. While the study presents minimal potential risks, if a participant’s results suggest potential susceptibility, this information will be confidentially discussed with their parents (only after obtaining explicit parental consent) to facilitate a referral for further assessment. This sensitive information will not be disclosed to school authorities. The psychologists assisting with data collection are prepared to identify and offer support for any immediate distress participants might experience.

Access to the final, anonymized dataset is carefully controlled, limited to the primary author, data analyst, principal investigator, and supervising committee members directly involved in data analysis and reporting. Importantly, there are no contractual limitations restricting the investigators’ access to the data. The findings from this research will be shared through various channels, including publications in peer-reviewed journals, presentations at academic conferences, and mandatory reporting on the ClinicalTrials.gov database. In addition, summaries might be shared with participants and other relevant stakeholders when appropriate. Authorship for publications will align with the established International Committee of Medical Journal Editors guidelines. Following the main dissemination of results, the research team will address reasonable requests for access to the complete study protocol, the anonymized participant dataset, and the statistical code used for analysis.

### Potential Risk to Participants

This study has minimal potential risks for the participants. While obtaining informed consent from the parents and assent from the participants, it will be made clear that information regarding those considered susceptible, based on their scores, will be shared with their parents, and they will be referred for further assessment after the parents’ consent. However, the status of the participants will be kept confidential from the school authorities. The study will involve psychologists who will conduct the interview along with the principal investigator; hence, they will be able to screen the potential participants.

### Ancillary and Posttrial Care and Referral Mechanism

The data will be collected by trained psychologists, and all the identified cases will be referred to a counselor who will also be hired for the study, so they can refer the extreme cases for further treatment. The employed counselors will be on hand at the study sites for data collection and intervention. The cases presenting extreme suicidal ideation thoughts will be referred for follow-up. The screening and referral expenses of the participants will be borne by the study [71]. The attendees will also be informed about the resources that are accessible, such as the hotlines of the suicide prevention organizations operating in Pakistan.

For this trial, the criteria for discontinuing or modifying allocated interventions for a participant will include a participant’s explicit request to withdraw, the emergence of any unforeseen serious adverse events related to the intervention, or if the participant’s mental state significantly deteriorates, requiring a different level of care. Strategies to improve adherence to the intervention protocol will involve clear communication of the program’s duration and expectations during the informed consent process; regular engagement with participants, which may include follow-ups; and ensuring the intervention is delivered in a manner that is acceptable and feasible for the participants. Participants will be informed about any permitted or prohibited concomitant care or interventions during the trial period to avoid any potential confounding factors.

## Results

### Study Progress to Date

As of July 30, 2025, significant progress has been made in the initial phases of the project. This includes the successful acquisition of permissions from the AKU-ERC and relevant district education offices in GB and intervention development and validation. Furthermore, the pilot testing of the intervention materials was completed in October 2024, providing valuable insights for the main study. Currently, the recruitment of participants is underway and is scheduled to commence fully in August 2025. Importantly, funding for this project has already been confirmed through a grant from the Fogarty International Center at the National Institutes of Health.

### Projected Timeline

This project is proceeding according to the subsequently mentioned timeline, which outlines the key milestones and their anticipated completion dates. Participant recruitment is expected to be completed by July 2025, followed immediately by baseline assessments, also in July 2025. The intervention delivery period is scheduled to run from July 2025 to August 2025. Postintervention assessments will take place in September 2025. Data cleaning and analysis will commence immediately following the postintervention assessments. Subsequently, manuscript preparation will begin, and the final phase will involve dissemination activities to share the study findings.

## Discussion

### Anticipated Findings

This study aims to evaluate the effectiveness of the RAAHI and safeTALK suicide prevention programs in improving the mental well-being of school-going adolescents in GB, Pakistan. This study’s predicted findings indicate that the suicide prevention program will considerably improve the mental health of school-going teenagers in GB. Following the intervention, it is anticipated that suicidal ideation and actions will decrease substantially, with fewer adolescents reporting self-harm inclinations and suicide attempts. Furthermore, mental health indicators, such as depression, anxiety, and stress levels, are expected to fall sharply, while resilience, coping skills, and emotional regulation among adolescents are likely to increase. The program is also projected to raise suicide prevention awareness by educating students about risk factors and existing support systems, resulting in increased help-seeking behavior.

A positive transformation in the school atmosphere is expected, with less stigma around mental health talks and increased peer-to-peer assistance. Furthermore, teachers and school personnel are expected to take part in detecting and managing mental health issues more actively, resulting in a more conducive learning environment. In the long run, the program has the potential to develop long-term mental health initiatives within schools, ensuring that suicide prevention measures are integrated into student support services. Overall, the intervention is expected to make the school community safer and more resilient, helping to reduce the risk of suicide among adolescents in the region.

The effectiveness of school-based suicide prevention programs has been well-documented in high-income countries, where structured interventions have demonstrated significant improvements in mental health literacy, help-seeking behaviors, and suicide risk reduction [75]. However, there is a significant gap in the implementation and evaluation of such interventions in LMICs, particularly in South Asia. Previous research highlights that culturally tailored and community-based interventions are more effective in addressing the unique sociocultural determinants of adolescent suicide [76]. This study builds upon this evidence by evaluating safeTALK, a globally recognized suicide prevention program that was adopted, and RAAHI, a locally developed intervention, within the unique context of GB. Unlike previous studies, which primarily focus on universal school-based interventions, this research also considers the role of peer-to-peer support and capacity-building among educators in creating a sustainable suicide prevention model [77].

Given the alarming rise in suicide rates in Pakistan and the absence of structured prevention initiatives, these findings will provide valuable insights into the potential impact of these interventions on suicidal ideation, suicidal awareness, suicide literacy, attitudes toward help-seeking, and mental health outcomes (depression, anxiety, and stress) among participants following the intervention. These findings align with previous research indicating that structured suicide prevention programs can enhance awareness, reduce stigma, and foster positive coping mechanisms among adolescents [78]. However, while previous studies have predominantly focused on Western contexts, this study highlights the effectiveness of such interventions in a culturally unique and resource-limited setting [79]. Notably, the increase in self-efficacy and adaptive coping strategies suggests that these programs may play a role in promoting long-term resilience among at-risk adolescents [80]. Interestingly, our results may reveal gender-specific response patterns that deviated from those observed in high income countries [79]. This divergence likely stems from the distinct cultural and social landscape of GB, Pakistan, where mental health stigma and traditional gender roles significantly impact how individuals seek assistance [81]. Furthermore, this research offers novel data, demonstrating the efficacy of suicide prevention strategies within resource-constrained, rural areas.

### Public Health Implications

As per the researcher’s knowledge, this research study will be the first to establish the effectiveness of a suicide prevention program in improving the mental well-being of school-going adolescents in Pakistan. The results of this research will assist in upscaling suicide prevention interventions tailored to the cultural and social context of GB. RAAHI and safeTALK are expected to bring a positive change in this cohort of adolescents. The success of the intervention will be that people of Pakistan, especially adolescents, start normalizing talking about this taboo topic. This will help in recognizing and identifying those in need of help in any community. Moreover, awareness regarding suicide may improve resilience and increase help-seeking behavior among adolescents. Ultimately, this preventive intervention program will contribute to reducing the burden of suicide in Pakistan.

### Future Directions

Future research in school-based suicide prevention, particularly in LMICs, should prioritize longitudinal studies to assess long-term impacts and expand to diverse populations, including out-of-school adolescents. Integrating digital mental health tools and developing gender-specific interventions will enhance scalability and effectiveness. Qualitative studies are vital to understanding cultural barriers and student experiences, guiding program refinement. Policy makers must implement national strategies combining school programs, peer support, and community engagement. Structured training programs, complemented by accessible professional care and community support, are essential for reducing stigma and promoting early intervention. Comparative studies of various prevention models will identify optimal approaches for LMICs.

### Dissemination

Peer-reviewed publications and conference presentations will be prioritized to engage academic and mental health professionals. In addition, collaboration with the Ministry of National Health Services Regulations and Coordination, government of Pakistan; education departments; and nongovernmental organizations will facilitate the translation of research findings into school-based mental health policies. A key component of the dissemination strategy will involve community outreach programs, including workshops for educators, parents, and students, to enhance suicide literacy and mental health awareness [82]. Finally, digital platforms, such as webinars and social media campaigns, will be used to increase public engagement and encourage discussions on youth suicide prevention in Pakistan.

### Strengths and Limitations

Because there is no standard intervention available for suicide prevention for adolescents in the Pakistani context, this research will assist in upscaling suicide prevention interventions tailored to the cultural and social context of GB among school-going adolescents in Pakistan. This study aims to bridge this gap by evaluating the effectiveness of suicide prevention programs specifically designed for Pakistani adolescents, thereby providing crucial data to inform national mental health strategies and policies. This study’s strong pre- and postintervention design enabled a clear evaluation of the program’s immediate effect on participants’ mental health. The use of established measurement instruments bolsters the dependability of our results.

However, due to time, budget, and logistical constraints, this feasibility study will only be conducted in 4 schools in GB, Pakistan, limiting potentially diverse implementation experiences and outcomes in rural or public-school settings. This may restrict the generalizability of the study findings. Moreover, using a quasi-experimental design without an appropriate control group can potentially undermine the internal validity of the study. Third, this study’s sample was limited to school-going adolescents, potentially restricting the generalizability of findings to out-of-school adolescents or other high-risk groups. In addition, the absence of a long-term follow-up limits our ability to determine the sustained impact of these interventions. However, reliance on self-reported data raises the possibility of response biases, particularly social desirability, which is significant given the prevailing mental health stigma in this area. Furthermore, while we acknowledge the potential influence of factors such as socioeconomic status, family support, and preexisting mental health issues, these were not fully controlled in our analysis. Future research should strive to include a broader range of variables to strengthen the study’s conclusions. Finally, it is important to note that the results of this investigation are specific to the studied context and may not be readily applicable to different cultural or geographic populations.

### Conclusions

This study aims to evaluate the impact of RAAHI and safeTALK, culturally adapted suicide prevention programs, on adolescents in GB across 8 weeks. By measuring changes in suicidal thoughts, knowledge, and confidence, the research will inform evidence-based strategies for researchers, clinicians, and policymakers. These programs present a viable approach to combat the rising suicide rates, improve mental health, and promote positive attitudes within the community. However, long-term success requires addressing systemic challenges to ensure these interventions are integrated into wider public health systems.

## Appendix

Multimedia Appendix 1 The Aga Khan University Ethics Review Committee approval.

Multimedia Appendix 2 Consent form.

Multimedia Appendix 3 Assent form.

## Abbreviations

AKU-ERC: Aga Khan University Ethics Review Committee
CVI: content validity index
DASS-21: Depression, Anxiety, and Stress Scale–21 Items
GB: Gilgit-Baltistan
LMIC: low- and middle-income country
MSSI: Modified Scale for Suicidal Ideation
RCT: randomized controlled trial
SPIRIT: Standard Protocol Items: Recommendations for Interventional Trials
TALK: tell, ask, listen, and keep safe

## Data Availability

The datasets generated or analyzed during this study are available from the corresponding author on reasonable request
