# Supplementary material for: Validation and Testing of a Suicide Prevention Program in Preventing Suicidal Ideation and Improving the Mental Well-Being of School-Going Adolescents: Protocol for a Pre-Post Intervention Study
Source: JMIR Res Protoc. 2025 Dec 12;14:e67193. doi: 10.2196/67193 (PMC12743240; doi:10.2196/67193)
Supplement: Multimedia Appendix 1 [file resprot_v14i1e67193_app1.pdf]

آغا خان یونیورسٹی
THE AGA KHAN UNIVERSITY

15-May-2023

Mrs. Yasmin Nadeem Parpio
Department of School of Nursing and Midwifery
Aga Khan University
Karachi

Dear Mrs. Yasmin Nadeem Parpio,

2023-8509-24844. Yasmin Nadeem Parpio: Validation and Testing of Suicide Prevention Program in improving mental well-being among school going Adolescents in GBC, Pakistan

Thank you for submitting your application for ethical approval regarding the above mentioned study.

Your study was reviewed and discussed in ERC meeting. There were no major ethical issues. The study was given an approval for a period of one year with effect from 15-May-2023. For further extension a request must be submitted along with the annual report.

List of document(s) approved with this submission.

Submission Document Name	Submission Document Date	Submission Document Version
CITI certificate Yasmin Nadeem Parpio	23-Mar-2022	1.7
Dr Tazeen- CITI Certificate	04-Apr-2023	1.7
Dr. Murad M Khan - Assurance training certificate	04-Apr-2023	1.7
citiCompletionReport10799077-UzmaRK	04-Apr-2023	1.7
citiCompletionCertificate RN 2021	05-Apr-2023	1
citiCompletionCertificate_DrNuruddin	05-Apr-2023	1
GCP Certificate-Salman Shahzad	05-Apr-2023	1
Affidavit for Translation-Suicide Project.docx (1) (signed)	10-Apr-2023	1
Consent Final English version 2023	10-Apr-2023	1
Final PhD Proposal Yasmin Nadeem Parpio-2023	11-Apr-2023	1
رضامندی فارم براےوالدین Consent Urdu Version	11-Apr-2023	1
Questionnaire English Final	11-Apr-2023	1
Permission Letter-AKESP	19-Apr-2023	1
Assent English Version 2023 Final.docx	19-Apr-2023	2
Final Assent Urdu Version.docx	19-Apr-2023	2
Final Questionnaire Urdu Version 2023.docx	19-Apr-2023	1

Any changes in the protocol or extension in the period of study should be notified to the Committee for prior approval. All informed consents should be retained for future reference.

Please ensure that all the national and institutional requirements are met.

Thank you.

Sincerely,

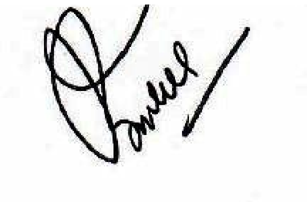

Dr. Bushra Moiz

Chairperson
Ethics Review Committee
