# Supplementary material for: Validation and Testing of a Suicide Prevention Program in Preventing Suicidal Ideation and Improving the Mental Well-Being of School-Going Adolescents: Protocol for a Pre-Post Intervention Study
Source: JMIR Res Protoc. 2025 Dec 12;14:e67193. doi: 10.2196/67193 (PMC12743240; doi:10.2196/67193)
Supplement: Multimedia Appendix 2 [file resprot_v14i1e67193_app2.pdf]

Informed Consent Form for Parents

**Project Title:** "Validation and Testing of Suicide Prevention Program in improving mental well-being among school going Adolescents in Karachi, Pakistan"	**ERC Num: 2023-8509**
**Supervisor**: Dr. Rozina Nuruddin<br>Assistant Professor, Community<br>Health Sciences, AKU Pakistan	**Contact details**: 02134864833<br>Ext 4833<br>Email rozina.nuruddin@aku.edu
**Principal Investigator:** Yasmin Nadeem Parpio	**Contact details**: 03155948771<br>Email yasmin.parpio@aku.edu
**Location of study:** Aga Khan University Hospital	**Sponsorship/Financial benefits:** NA

OVERVIEW

"I am Yasmin Parpio, PhD student, Aga khan University, Karachi, Pakistan, conducting a research study on "Validation and Testing of Suicide Prevention Program in improving mental well-being among school going Adolescents in Northern, Pakistan"

"This study aims to validate and evaluate the efficacy of a suicide prevention program in reducing suicidal ideation, increasing knowledge and confidence in discussing suicide, and improving adolescents' readiness to seek help in Northern, Pakistan. You will be provided with all the required information regarding the study. Moreover, before making a decision, you can ask anything for clarification that makes you feel uncomfortable with this study. In addition, your child has the right to withdraw from the study at any time, and not participating in this study will not affect you at any cost.

PURPOSE OF THIS RESEARCH STUDY

This study purpose is to validate and evaluate the efficacy of a suicide prevention program in reducing suicidal ideation, increasing knowledge and confidence in discussing suicide, and improving adolescents' readiness to seek help in Northern Pakistan.

PROCEDURE

A Quasi experimental study design will be used to assess the effectiveness of Suicide Prevention Program and assessment of their health seeking behavior before and after giving intervention.

Study Setting

The study will be conducted in three schools located in Hunza, GBC province, run under Aga Khan Education Service, Pakistan (AKESP). These are:

1. Aga Khan Secondary School, Ghizer
2. Aga Khan Secondary School, Karimabad Hunza
3. Aga Khan Secondary School, Gilgit

Our study will invite students studying in grades 9 and10.

If you let your child to participate, in this study, your child's participation will be depending on their availability. Before participating in this study, your child will sign written assent form. The data will be collected as per your child's preference of language, i.e., English or Urdu in three times. The data will be collected as per of your child's convenience and comfort level. It will last approximately 30-40 minutes. This is a culturally relevant program as it applies basic 'TALK' steps (Tell, Ask, Listen, and Keep Safe); and connect the suicidal person with suicide first aid help. The data collector or moderator is responsible for getting training from the Living Works organization so that he/she will be enabled for the intervention. The initial session will be of 2 hours, and it will help your child to improve their suicide literacy. A pocket card containing the **'suicide alert steps'** is also provided to your child at the end of the training. The second workshop will introduce the safeTALK program which will be of 3 hours. The training includes presentations, video, discussion, questions and role-play. The intent of this training is to reduce suicidal ideation and suicide among the population and improve their confidence and help seeking behavior. This program intends to cater adolescents who are aged 15 years and older. The safe TALK workshops will be delivered to adolescents, in the form of 1 day workshop approximately 4 hours. Each student will attend one workshop only. Researcher along with psychologist will administer this training with the help of video and material prepared by Living Works. Firstly, the psychologist along with the researcher will perform risk assessment of the students for suicidal ideation and suicidal behavior. Secondly, psychologist will administer training along with researcher which consists of video and material prepared by Living Works.

POTENTIAL BENEFITS:

The potential benefit may be improvement in mental wellbeing. Many studies have already proved that Safe talk intervention improve mental wellbeing. The results of this research will assist in upscaling contextualized suicide prevention intervention. Safe-TALK tends to bring positive change in this cohort of adolescents. The success of the intervention will be that people especially adolescents start normalizing talking about this taboo topic. This will help to recognize and identify those in need of help around us. The awareness regarding suicide may improve resilience and increase help-seeking behavior among adolescents. Ultimately, this preventive intervention program will contribute to reducing the burden of suicide in Pakistan

POSSIBLE RISKS OR DISCOMFORT

Participating in this study poses little to no risk to your child, and we will take all necessary steps to ensure their comfort throughout the process. Your child's status will be kept confidential from school authorities, and the psychologists involved in the study will conduct interviews under the supervision of the principal investigator. This will enable them to screen potential participants effectively.

FINANCIAL CONSIDERATIONS

This study will offer transport charges to you.

CONFIDENTIALITY

We have implemented specific measures to guarantee the confidentiality of your child's information. Instead of using names, we will assign codes to the participants. Furthermore, all data will be securely locked and accessible only to the principal investigator and core team responsible for the thesis.

RIGHT TO REFUSE OR WITHDRAW

Your child will have the right to withdraw from study at any point in time. They will not be forced. Your child has the right to refuse to take part in study.

DISSEMINATION OF RESULTS

The study's findings will be disseminated to program implementers and policymakers. While the results, along with other data, may be published for scientific purposes, your child's identity will remain confidential and unidentifiable. However, the ethics review committee of Aga Khan University may inspect any records or data obtained from your child's participation in the study

AVAILABLE SOURCES OF INFORMATION

In case of further questions or queries about the study, or the consent form, you may contact the research investigator (myself) Yasmin Parpio at Aga Khan University, Karachi, Pakistan (03155948771) or Dr. Rozina at AKU-SONAM, Karachi, Pakistan (02134864833).

AUTHORIZATION

I have read the consent form and understand the study being described. I have had an opportunity to ask questions and those questions have been answered. I am free to ask questions about the study in the future. I freely consent to let my children participate in the research study, understanding that I may discontinue participation at any time without penalty. A copy of this consent form has been made available to me

**Name of Participant**	**Name of Principal Investigator:**	**Name of the person obtaining consent: ____________**
**Name of the parent Signature: __________________** **Date: _________________**	**Signature: ___________** **Date: ________________**	**Signature: _____________** **Date: _________________**

For participants parents who cannot read

WITNESS:

I have witnessed the entire reading process, on behalf of the participant. The questions of the participants are answered to satisfaction, by the thesis core team. Therefore, I endorse that the participant has voluntarily agreed to participate in the current study

**For Participants’ Parents who are unable to read**
Name of the person Obtaining Consent ___________________________________
Signature: ___________________________Date: _________________________
Parent Thumb Print: _____________Witness Name: ________________
