# Supplementary material for: Validation and Testing of a Suicide Prevention Program in Preventing Suicidal Ideation and Improving the Mental Well-Being of School-Going Adolescents: Protocol for a Pre-Post Intervention Study
Source: JMIR Res Protoc. 2025 Dec 12;14:e67193. doi: 10.2196/67193 (PMC12743240; doi:10.2196/67193)
Supplement: Multimedia Appendix 3 [file resprot_v14i1e67193_app3.pdf]

Assent Form for Participants

**Project Title:** Title: **Validation and Testing of Suicide Prevention Program in improving mental well-being among school going Adolescents in Northern Pakistan**	**ERC Num: 2023-8509**
**Supervisor**: Dr. Rozina Nuruddin<br>Assistant Professor, Community<br>Health Sciences, AKU Pakistan	**Contact details**: 02134864833<br>Ext 4833<br>Email: rozina.nuruddin@aku.edu
**Investigator:** Yasmin Nadeem Parpio	**Contact details**: 03155948771<br>Email: yamin.parpio.aku.edu
**Location of study:** Aga Khan University Hospital	**Sponsorship/ Financial benefits:** NA

OVERVIEW

I am Yasmin Nadeem Parpio, a PhD student from Aga Khan University in Karachi, Pakistan. I am conducting a research study on “Validation and Testing of Suicide Prevention Program in improving mental well-being among school going Adolescents in Gilgit Baltistan. This study aims to evaluate the effectiveness of this program in reducing suicidal thoughts and increasing knowledge and confidence in discussing suicide. It also aims to improve the readiness of adolescents to seek help. You will be provided with all the necessary information about the study, and you can ask any questions before making a decision. You have the right to withdraw from the study at any time, and not participating in this study will not have any negative impact on you.

PURPOSE

The aim of this study is to test and measure how effective a suicide prevention program is in reducing suicidal thoughts, increasing understanding and confidence in discussing suicide, and improving adolescents' readiness to seek help in Gilgit Baltistan.

PROCEDURES

This study will use a Quasi-experimental study design to evaluate the effectiveness of the Suicide Prevention Program. It will also assess the health-seeking behavior of participants before and after receiving the intervention.

The study will be conducted in three schools located in Gilgit Baltistan run under Aga Khan Education Service, Pakistan (AKESP). These are:

1. Aga Khan Secondary School, Ghizer
2. Aga Khan Secondary School, Karimabad Hunza
3. Aga Khan Secondary School, Gilgit

Our study will invite students studying in grades 9 and10.

Your participation in this study depends on your availability, and I invite you to participate by signing a written consent form. Data will be collected in either English or Urdu, according to your language preference, and will take approximately 30-40 minutes at your convenience and comfort level. This program is culturally relevant and follows the basic 'TALK' steps (Tell, Ask, Listen, and Keep Safe) to connect suicidal individuals with first aid help. The data collector or moderator has received training from the Living Works organization to intervene effectively. Participants will also receive a pocket card outlining the 'suicide alert steps' at the end of the training. Adolescents will attend a one-day workshop lasting approximately four hours, and the psychologist and researcher will conduct a risk assessment for suicidal ideation and behavior prior to administering the training using material prepared by Living Works.

POTENTIAL BENEFITS

By participating in this study, participants may experience a decrease in stress and anxiety levels, as previous studies have shown that the Safe-TALK intervention improves mental wellbeing. The results of this research will help to expand suicide prevention interventions that are relevant to the local context. Successful implementation of Safe-TALK can encourage open discussions about suicide, leading to better identification and recognition of those in need of help. This increased awareness may improve resilience and encourage adolescents to seek help

when needed. Ultimately, this preventive intervention program may contribute to reducing the burden of suicide in Pakistan.

POSSIBLE RISKS OR DISCOMFORT

There are minimal to no potential risks associated with participating in this study, and participant comfort will be ensured at every level. The confidentiality of participants will be maintained, and their status will not be disclosed to school authorities. Psychologists will be involved in the study and will assist the principal investigator in screening potential participants

FINANCIAL CONSIDERATIONS

This study will offer transport charges to the parents of the participants.

CONFIDENTIALITY:

We have taken concrete steps to ensure that your information remains confidential. Participants will be provided with codes instead of their names, and all data will be kept in a secure location with restricted access to only the principal investigator and thesis core team.

RIGHT TO REFUSE OR WITHDRAW

Participants will have the right to withdraw from study at any point in time. They will not be forced. Participants have the right to refuse to take part in study.

DISSEMINATION OF RESULTS

The results obtained from this study will be shared with program implementers and policymakers. The study's findings, including other data, may be published for scientific purposes, but no identifiable information about participants, including names, will be included. However, any records or data obtained as a result of participation in this study may be reviewed by the ethics review committee of Aga Khan University.

AVAILABLE SOURCES OF INFORMATION

In case of further questions or queries about the study, or the consent form, you may contact the research investigator (myself) Yasmin Parpio at AKU-SONAM, Karachi, Pakistan **(**03155948771) or Dr. Rozina at AKU-SONAM, Karachi, Pakistan (02133865472)

AUTHORIZATION

I have read the assent and understand the study being described. I have had an opportunity to ask questions and those questions have been answered. I am free to ask questions about the study in the future. I freely consent to let my children participate in the research study, understanding that I may discontinue participation at any time without penalty. A copy of this consent form has been made available to me

**Name of participant**<br>____________________<br>**Signature:**<br>____________________<br>**Date:** ____________________	**Name of Principal Investigator:**<br>____________________<br>**Signature:**____________<br>**Date:** ____________________	**Name of the person obtaining consent:**_____________<br>**Signature:**______________<br>**Date:** ____________________
